# Microarray data mining using Bioconductor packages

**DOI:** 10.1186/1753-6561-3-S4-S9

**Published:** 2009-07-16

**Authors:** Haisheng Nie, Pieter BT Neerincx, Jan van der Poel, Francesco Ferrari, Silvio Bicciato, Jack AM Leunissen, Martien AM Groenen

**Affiliations:** 1 Animal Breeding and Genomics Centre, Wageningen University, Marijkeweg 40, P.O. Box 338, 6700 AH, Wageningen, The Netherlands; 2 Laboratory of Bioinformatics, Wageningen University, Dreijenlaan 3, P.O. Box 569, 6700 AN, Wageningen, The Netherlands; 3 Department of Biology, University of Padova, Via G. Colombo 3, 35121, Padova, Italy; 4 Department of Biomedical Sciences, University of Modena and Reggio Emilia, via Campi 287, 41100, Modena, Italy

## Abstract

**Background:**

This paper describes the results of a Gene Ontology (GO) term enrichment analysis of chicken microarray data using the Bioconductor packages. By checking the enriched GO terms in three contrasts, MM8-PM8, MM8-MA8, and MM8-MM24, of the provided microarray data during this workshop, this analysis aimed to investigate the host reactions in chickens occurring shortly after a secondary challenge with either a homologous or heterologous species of *Eimeria*. The results of GO enrichment analysis using GO terms annotated to chicken genes and GO terms annotated to chicken-human orthologous genes were also compared. Furthermore, a locally adaptive statistical procedure (LAP) was performed to test differentially expressed chromosomal regions, rather than individual genes, in the chicken genome after *Eimeria *challenge.

**Results:**

GO enrichment analysis identified significant (raw p-value < 0.05) GO terms for all three contrasts included in the analysis. Some of the GO terms linked to, generally, primary immune responses or secondary immune responses indicating the GO enrichment analysis is a useful approach to analyze microarray data. The comparisons of GO enrichment results using chicken gene information and chicken-human orthologous gene information showed more refined GO terms related to immune responses when using chicken-human orthologous gene information, this suggests that using chicken-human orthologous gene information has higher power to detect significant GO terms with more refined functionality. Furthermore, three chromosome regions were identified to be significantly up-regulated in contrast MM8-PM8 (q-value < 0.01).

**Conclusion:**

Overall, this paper describes a practical approach to analyze microarray data in farm animals where the genome information is still incomplete. For farm animals, such as chicken, with currently limited gene annotation, borrowing gene annotation information from orthologous genes in well-annotated species, such as human, will help improve the pathway analysis results substantially. Furthermore, LAP analysis approach is a relatively new and very useful way to be applied in microarray analysis.

## Background

The summary paper [1] has introduced the microarray data and the biological background of the microarray experiment, in this analysis, we focus our analysis on the gene lists from three contrasts: MM8-PM8, MM8-MA8 and MM8-MM24. Each contrast has both up- and down-regulated significant gene list (FDR < 0.05), in total six gene lists were used for Gene Ontology [2] term enrichment analysis. The analysis in this paper was carried out using a number of different Bioconductor [3] packages (release version: BioC 2.3), GOstats [4], AnnotationDbi [5], and biomaRt [6]. Package Gostats uses hypergeometic test to identify significantly enriched GO terms in gene lists of interest.

Package GOstats also provides conditional hypergeometric test which uses the relationship among GO terms to decorrelate the results. Package AnnotationDbi Provides an interface and database connection code for annotation data packages using SQLite data storage, the annotation data packages were needed for GOstats package. Package biomaRt provides an R interface to BioMart databases [7]. To investigate the effects of different sources of microarray probe annotation on GO term enrichment analysis, two analyses were carried out: one used chicken gene information and the other one used chicken-human orthologous gene information.

Furthermore, a locally adaptive statistical procedure (LAP) [8] was performed to test differentially expressed chromosomal regions, rather than individual genes, in the chicken genome after *Eimeria *challenge. LAP is a non-parametric model-free statistical method for the identification of differentially expressed chromosomal regions, which accounts for variations in gene distance and density. The method is based on the computation of a standard statistic (e.g. SAM *t*-statistic) as a measure of the difference in gene expression patterns between groups of samples. The LAP analysis approach is a relatively new and interesting way of analyzing microarray data.

## Methods

### Chicken 20 k oligo array annotation

An updated chicken 20 k oligo-array annotation based on Ensembl [9] release 50 was downloaded from EADGENE Oligo Set Annotation Files homepage [10]. Human orthologous genes, if identified, were mapped to the corresponding chicken oligo probes present on the chicken array. The human Ensembl gene IDs were then used to extract human Entrez gene IDs via the Bioconductor package biomaRt by querying to the Ensembl genome database. The resulting human Entrez gene IDs were subsequently used to build a customized chicken array annotation R package using AnnotationDbi.

### GO enrichment analysis 

A GO term enrichment analysis was carried out using package GOstats and a conditional hypergeometric test algorithm provided within GOstats package was applied to each gene list. The conditional hypergeometric test will identify a GO term as significant if there is evidence beyond that provided by its significant children. The threshold for significance of the hypergeometric test was raw p-values < 0.05. Only GO terms in the category Biological Process (BP) were used in this analysis. Those GO terms were excluded from the result list when Count equal to 1 Or Size equal to 1, i.e. only 1 gene in the DE gene list links to this specific GO term or only 1 gene on the whole array links to this specific GO term.

### Differentially expressed chromosomal regions

Differentially expressed chromosomal regions were identified using locally adaptive procedure (LAP). LAP analysis was performed in R [11] and the threshold used in this analysis is q-values < 0.01, where q-value is the false discovery rate calculated from p-values between two group comparisons, i.e. p-values derived from each contrast.

## Results and discussion

### GO term enrichment analysis

All the GO enrichment analysis results are available in the Additional file 1 and Additional file 2. Here we will focus only on the selected GO terms related to immune response (see Additional file 1) to explain the three contrasts, MM8-PM8, MM8-MA8, and MM8-MM24.

#### (1) MM8-PM8 contrast

Genes that are up-regulated in the MM8-PM8 contrast show an enrichment of GO terms like, "immune response-activating cell surface receptor signalling pathway", "proteolysis involved in cellular protein catabolic process" and "focal adhesion formation". These terms all indicate that the chickens show primary immune responses at 8 hours after PM challenge.

Genes that are down-regulated in the MM8-PM8 contrast show an enrichment of GO terms like, "regulation of B cell differentiation", "regulation of T cell activation", "T cell selection" and "regulation of interferon-gamma biosynthetic process", terms indicative for a secondary immune response at 8 hours after homologous MM challenge.

These results clearly show the induction of different immune responses (primary vs. secondary) in chicken that encountered an *Eimeria *infection for the first time and chicken that had gone through an *Eimeria *infection at an earlier time in their life.

#### (2) MM8-MA8 contrast

No major differences on immune response related GO terms were identified in the MM8-MA8 contrast, these results show that, heterologous challenge MA triggers a very similar immune response as MM. Interestingly, the genes up-regulated in the MM8-MA8 contrast show an enrichment of GO term like "cell death" and "apoptosis", suggesting that the heterologous challenge caused more severe lesions in the chickens as compared to an homologous challenge.

No evidence shows that MM8 and MA8 trigger different immune responses in chicken, although the enriched GO terms indicate a more severe pathogenesis in case of heterologous challenge.

#### (3) MM8-MM24 contrast

As described in the MM8-PM8 contrast result, the homologous challenge already triggered a secondary immune response at 8 hours. No significant GO terms related to secondary immune response were found in MM8-MM24 contrast. The up-regulated genes in MM8-MM24 have enriched GO terms like "positive regulation of NF-kappaB transcription factor activity", and the down-regulated genes in MM8-MM24 have enriched GO terms like, "T cell receptor signalling pathway" and "interleukin-2 production". NF-kappaB is a key regulator of several important immune-related pathways, this suggests that immune response activators were already highly up-regulated at 8 hours compared to 24 hours and that a secondary immune responses kept on increasing from 8 hours to 24 hours after homologous challenge with MM.

#### Multiple testing problem

We have applied "BH" FDR control method for correction for multiple testing using R package multtest [12] and found only a few significant GO terms after correction (data not shown). In this analysis we used threshold of raw p-value < 0.05, the major reasons of not using the FDR control methods are (a) the structure of the GO graph is in conflict with the assumption of independence for the test and (b) multiple testing correction methods do not change the overall ranks of the results, using raw p-value at cut-off would still identify the relative important GO terms in the results.

#### Annotation Sources comparison

**Figure 1:**
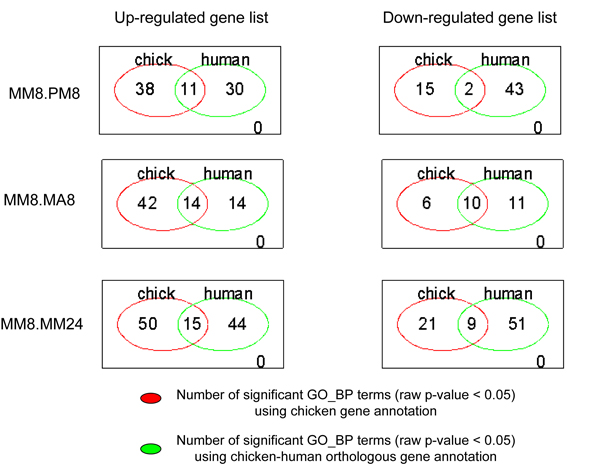
**Comparison of GO term enrichment analysis results**. Overlap of significantly enriched GO terms (raw p-value < 0.05) between the uses of chicken gene information versus chicken-human orthologous gene information.

In this section, GO enrichment analysis results using chicken gene annotation and chicken-human orthologous gene annotation are compared. All the GO term enrichment analysis results of this comparison are available in the Additional file 2 and Additional file 3. The overlap of the results of the GO term enrichment analysis using the chicken gene information and using the chicken-human orthologous gene information is shown in Figure 1. The overlap of the significant GO terms identified by both annotation sources is limited. Enriched GO terms using chicken genes and using chicken-human orthologous genes, as described above, gave a reasonably good insight of the underlying biological processes in this the experiment. The enriched GO terms based on the chicken annotation directly didn't reveal much detail in the ongoing processes after either homologous challenge or heterologous challenge (see Additional file 2). The enriched GO term using the chicken-human orthologous gene information had a higher power to detect significant GO terms (see Additional file 3), which can be explained by the higher coverage of annotation (GO terms) using this approach.

Performing the GO enrichment analysis using chicken-human orthologous genes, on one hand, extensively increased the coverage of the gene annotation of this chicken oligo array platform. Consequently, this increases the power of the hypergeometric test by having more annotated genes in the DE gene lists. On the other hand, care has to be taken by using this approach, as human and chicken are evolutionarily far apart. Therefore, some of the chicken-specific immune response processes may not be identified using this approach. Nevertheless, this approach helps researchers working with farm animals, e.g. chicken, to increase the biological insight from their microarray data by using human orthologous gene information.

#### Differentially expressed chromosomal regions

**Figure 2:**
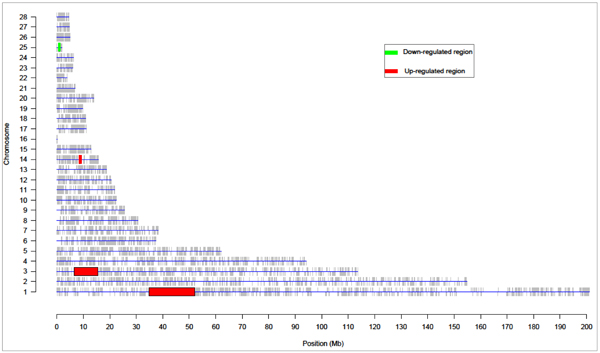
**Differentially expressed chromosomal regions for contrast MM8-PM8**. This figure showed the differentially expressed chromosomal regions for MM8.PM8 contrast (q-value < 0.01). In total three regions were up-regulated and one region was down-regulated. Red showed the up-regulated chromosomal regions, and Green showed the down-regulated regions.

Instead of testing enrichment of GO terms, chromosomal locations could be used as "annotation" to test whether certain chromosomal locations are more actively expressed than other regions. In this analysis, the differentially expressed chromosomal locations were identified using locally adaptive procedure (LAP). In total, three significant regions were up-regulated and one region was down-regulated comparing PM and MM infections (see details of those regions in Figure 2 and Additional file 4). No significant regions were identified in other contrasts. The identified differentially expressed chromosomal regions indicate that some of the co-localized genes are co-regulated during homologous challenge by MM, this region-wide gene expression regulation mechanism was reported in several other species [13,14].

## Conclusion

The GO term enrichment analysis provided a good insight in the biological processes involved in the *Eimeria *infection experiments. The GO enrichment analysis using several bioconductor packages described in this paper provides a practical, yet powerful, way of analyzing microarray data. Furthermore, the results suggest that using chicken-human orthologous gene information provides better insight in the biological processes underlying this specific microarray experiment than by using the annotation of chicken genes alone. This approach will be a helpful general method for researchers working with microarray data in species with less well annotated-genomes, like those of farm animals. Furthermore, LAP analysis approach is a relatively new and very useful way to be applied in microarray analysis to identify differentially expressed chromosomal regions under specific experimental conditions.

## List of abbreviations used

DE: Differentially Expressed; FDR: False Discovery Rate; GO: Gene Ontology; GO_BP: Gene Ontology Biological Process; PM: PBS-E. Maxima; MM: E. maxima-E. Maxima; MA: *E. maxima-E. acervulina*; LAP: locally adaptive statistical procedure

## Competing interests

The authors declare that they have no competing interests.

## Authors' contributions

HN analyzed the data and drafted the manuscript, all other authors helped to improve the manuscript. PBTN and JAML helped with the re-annotation of the array, JP and MAMG contributed to the biological interpretation of the results, FF and SB performed the analysis of differentially expressed chromosomal regions. All authors read and approved the final manuscript.

## Supplementary Material

Additional file 1 **GO enrichment analysis results with selected immune related GO terms**. This table shows GO enrichment results with selected GO_BP terms. (For contrasts MM8.PM8 and MM8.MM24 results, only immune-related GO_BP terms which have at least two genes linked to each one of them were included). Click here for file

Additional file 2 **GO term enrichment results (raw p-value <0.05) using chicken genes**. This table shows the GO enrichment analysis results using chicken gene information. Click here for file

Additional file 3 **GO term enrichment results (raw p-value < 0.05) using chicken-human orthologous genes**. This table shows the GO term enrichment analysis results using chicken-human orthologous genes information. Click here for file

Additional file 4 **Differentially expressed chromosomal regions for MM8-PM8 contrast**. This table shows the chromosomal locations of three up-regulated chromosomal regions and one down-regulated chromosomal region for MM8-PM8 contrast. Click here for file
